# Relationships between Thermoplastic Type and Properties of Polymer-Triticale Boards

**DOI:** 10.3390/polym11111750

**Published:** 2019-10-25

**Authors:** Radosław Mirski, Pavlo Bekhta, Dorota Dziurka

**Affiliations:** 1 Department of Wood-Based Materials, Poznań University of Life Sciences, 60-627 Poznań, Poland; dorota.dziurka@up.poznan.pl; 2 Department of Wood-Based Composites, Cellulose, and Paper, Ukrainian National Forestry University, 79057 Lviv, Ukraine; bekhta@nltu.edu.ua

**Keywords:** polymer-triticale boards, thermoplastic polymers, straw, hydrophobicity, mechanical properties

## Abstract

This study examined the effects of selected types of thermoplastics on the physical and mechanical properties of polymer-triticale boards. The investigated thermoplastics differed in their type (polypropylene (PP), polyethylene (PE), polystyrene (PS)), form (granulate, agglomerate) and origin (native, recycled). The resulting five-ply boards contained layers made from different materials (straw or pine wood) and featured different moisture contents (2%, 25%, and 7% for the face, middle, and core layers, respectively). Thermoplastics were added only to two external layers, where they substituted 30% of straw particles. This study demonstrated that, irrespective of their type, thermoplastics added to the face layers most favorably reduced the hydrophobic properties of the boards, i.e., thickness, swelling, and V100, by nearly 20%. The bending strength and modulus of elasticity were about 10% lower in the experimental boards than in the reference ones, but still within the limits set out in standard for P7 boards (20 N/mm^2^ according to EN 312).

## 1. Introduction

Wood plastic composites (WPCs) are usually made from forestry waste, including waste wood flour, wood shavings, and sawdust, and can be produced using different methods, e.g., extrusion, injection or compression molding [1]. Extrusion is a predominant technology for the manufacturing of WPCs, even though it only allows for predetermined dimensions of the boards. Another possibility is to produce WPCs on a flat-press [2,3,4,5,6,7,8], similarly as in the industrial particleboard manufacturing process. This is a new, technically more advantageous, and simple method for producing large-size WPC boards of different densities. It offers higher productivity at relatively low-pressure requirements, creates a positive environmental image of wood as a renewable material, enables the saving of wood raw material due to the lower density of composite boards, and lowers the production costs as compared with other methods [3]. Moreover, the resulting products closely compare to commercial medium-density fiberboard (MDF), particleboard, oriented strand board (OSB), and plywood.

The physical and mechanical properties of WPCs largely depend on the type of thermoplastic used, content, and properties of wood fillers, pressing parameters, and wood–plastic interactions [9,10,11,12,13,14]. The literature contains many reports on manufacturing WPCs that account for the type of thermoplastic, particle size, and influence of various technological factors [9,10,11,12,13,14].

Typical WPCs (thermoplastic content 20%–60%, wood particle content 40%–80%) are manufactured by extrusion or injection. They can also be produced using pressure methods similar to traditional board manufacturing technology involving cyclic or continuous pressing [8,15]. Additionally, chip-polymer flat-pressed composite WPCs are favorably produced by layering their mat, i.e., alternating the layers of ground thermoplastic materials and lignocellulosic particles prior to pressing [16]. Borysiuk et al. [17] demonstrated the advantages of using particles of similar shapes as this minimizes their sorting during sieving. Some authors [18,19] suggested that a reduction in the size of particles could improve the properties of particleboards made from agricultural fibers. In another work [20], binderless boards were successfully manufactured from rice straw powder with a particle size smaller than 1 mm. However, the flat-pressing method of polymer-lignocellulosic composite production requires a long pressing time and/or high temperature of the heating plates to overheat the mat and ensure thermoplastic softening. For this reason, it is better to use thermoplastics only in the layers where they can be easily melted. An important advantage of lignopolymer composites is their higher resistance to water as compared with other solid wood and wood-based materials. The polymer mechanically blocks the access of water to wood particles [21].

The demand of the wood industry—including the producers of wood-based materials—for lignocellulosic materials in the coming years will only be possible to satisfy by using the existing potential reserves. The source of additional raw material for production of wood-based materials should be plantations of fast-growing trees and annual plants. In the latter case, the board industry may use such plants and plant products as grasses, reeds, cereal straw or stems, and leaves of nettles. It also seems favorable to use commonly cultivated annual plants to minimize the risk of lignin–cellulose biomass supply. Despite the seasonality of supply, necessity of storage, low bulk density, and other negative aspects of using annual plants in board manufacturing, they still should be considered as raw materials of full value [22,23,24,25]. Cereal straw as a renewable raw material is one of the most important agricultural wastes generated in huge quantities in many countries of the world. The worldwide production of cereal straw is estimated at 1.5 billion m^3^ annually [26]. The countries of the European Union produce about 140 million tons annually, of which only 2%–3 % is used by the industry as a raw material for WPCs [27]. The straw is first ground into a powder that forms a kind of reinforcement for the polymer matrix. Many studies have been carried out on the utilization of annual plant fibers in lignocellulosic composites, such as particleboard, medium-density fiberboard (MDF) and WPCs [19,21,22,26,28,29,30,31,32,33,34]. A review paper by Abba et al. [35] presented different methods and procedures for the production of plastic composites from agricultural waste materials. In general, utilization of lignocellulosic fibers in wood-based composites offers several advantages, such as recyclability, fewer health hazards for the operators, low density, greater deformability, less abrasiveness to equipment, biodegradability, and low cost. Their major drawback involves relatively poor compatibility with hydrophobic thermoplastics that often results in poor mechanical properties [36].

Virgin thermoplastics, such as polypropylene (PP), polyethylene (PE), polystyrene (PS), and polyvinyl chloride (PVC), are commonly used as matrices for manufacturing WPCs [37] with wood flour, but using recycled plastic as a matrix was only reported in a few studies [6,37]. A major portion of global municipal solid waste includes post-consumer plastic materials, like high-density polyethylene (HDPE), low-density polyethylene (LDPE), and PVC, which can potentially be used in WPC manufacturing. For example, plastic film is widely used for packaging daily necessities, causing a serious problem for the environment. Therefore, utilizing waste plastic is important from the perspective of environmental protection and recycling. 

Considering the above, it seems that annual plant straw combined with thermoplastics could be used for manufacturing wood-based materials with high moisture resistance. For this reason, the aim of this study was to examine the effects of selected types of thermoplastics on physical and mechanical properties of polymer-triticale boards.

## 2. Materials and Methods

**Figure 1:**
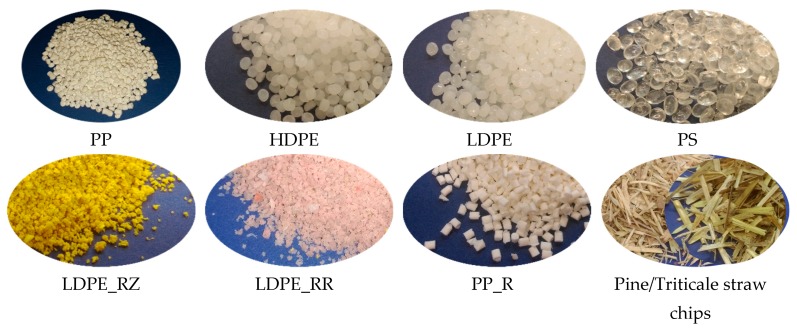
The photographs of the fillers.

**Figure 2:**
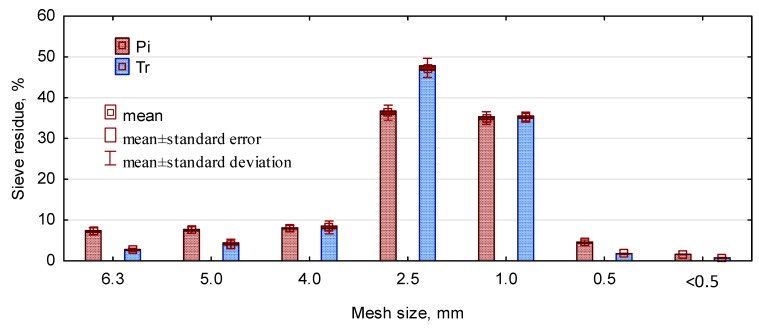
Sieve analysis of lignocellulose particles used in the study, (Pi—pine chips, Tr—triticale straw chips).

The study involved triticale straw and pine chips. The straw, obtained in agricultural bales, was ground in a laboratory mill into particles of dimensions as close as possible to pine chips. The linear dimensions of triticale particles were 17.8 mm in length, 2.55 mm in width, 0.30 mm in thickness, and for the pine chips, they were 13.73, 2.03, and 1.04 mm, respectively. A detailed dimensional analysis is presented in our previous publication [25]. The pine chips were industrial chips intended for core layers of three-ply P2 boards. Visual inspection confirmed disparate morphology of the fillers. The fillers are shown in Figure 1. The pre-selected material was sorted on a sieve with a mesh size of 0.5 mm in order to separate fine particles and silty fractions. Figure 2 presents the results of the sieve analysis. 

We produced five-ply boards with the following layer share: 0.5:0.5:1:0.5:0.5. The layers differed in the type of material and moisture content:

➢ The face layers (1, 5) were made from straw with the moisture content of 2%;
➢ The intermediate layers (2, 4) from straw with the moisture content of 25%;
➢ The core layer (3) from chips with the moisture content of 7%.

The effectiveness of a mat pressing into a board depends on numerous factors associated with the mat (raw material moisture content, degree of fineness, adhesive share) and pressing parameters (temperature, pressure, time) [38,39]. The time in which the internal layers reach the temperature of 100–110 °C largely depends on the mat moisture content [40], which is why the external layers are commonly made from materials of a higher moisture content. However, a lower moisture content finally allows for achieving a higher temperature that is crucial when using refractory additives during the mat formation. Some authors [41,42,43] indicate that polymers most effectively melt with lignocellulosic material when the moisture content of the material is below 3%–4%. For these reasons, our experimental boards consisted of the polymer containing layers of low moisture content, intermediate layers with high moisture content that facilitated heat transfer inside the mat, and a core layer with standard moisture content.

The study involved the following thermoplastics differing in the type, form, and softening point (Table 1):

- PP—polypropylene impact copolymer (Zakład Przetwórstwa Tworzyw Sztucznych, Czapury, Poland);
- HDPE—high-density polyethylene (Basell Orlen Polyolefins Sp. z o.o., Płock, Poland);
- LDPE—low-density polyethylene (Basell Orlen Polyolefins Sp. z o.o., Płock, Poland);
- PS—polystyrene (Zakład Przetwórstwa Tworzyw Sztucznych, Czapury, Poland);
- LDPE rec. yellow—low-density polyethylene from recycled scraps of yellow plastic (Zakład Przetwórstwa Tworzyw Sztucznych, Czapury, Poland);
- LDPE rec. pink—low-density polyethylene from recycled scraps of pink plastic (Zakład Przetwórstwa Tworzyw Sztucznych, Czapury, Poland);
- PP recycled—polypropylene from reusable packaging (Zakład Przetwórstwa Tworzyw Sztucznych, Czapury, Poland).

The thermoplastics were added to layers 1 and 5, where they substituted 30% of straw particles. The reference boards followed the same moisture content and particle type pattern but without polymers in 1 and 5 layers. Irrespective of the layer, the lignocellulosic material was glued with pMDI in the amount of 4% of dry weight of the adhesive per dry weight of the particles. The mat was formed manually to produce 15 mm-thick boards of the target density 600 kg/m^3^. The heating plate temperature was 200 °C, unit pressure 2.5 MPa, and pressing time 20 s per mm of board thickness. The mat was pressed between metal plates. Following hot pressing, the molds, together with the metal plates, were transferred onto a cold press, where they were kept under 1000 N load until their temperature dropped below 80–100 °C. The temperature was monitored with K-type thermocouples attached to the upper and lower surfaces of the plates. For each variant, we produced three molds 700 mm × 450 mm in dimension.

After conditioning (seven days, 55 ± 5% RH, 21 ± 1 °C), the boards produced this way were tested as per relevant standards and the following parameters were assessed:

- bending strength (MOR) and modulus of elasticity (MOE) according to EN 310 [44];
- internal bond (IB) according to EN 319 [45];
- internal bond after the boiling test (V-100) according to EN-1087-1 [46];
- thickness swelling (TS) after 24 h according to EN 317 [47] and water absorption (WA).

The assessments of mechanical properties and water resistance involved from 10 to 16 samples of each variant, and the remaining analyses were made in three to five replications. The results were analyzed using the STATISTICA 13.1 package (StatSoft Inc., Tulsa, OK, USA), and uncertain measurements were discarded prior to in-depth statistical analysis. The analysis was based on ANOVA, and homogeneous groups were tested with HSD Tukey′s test (HSD) and the Bonferroni test. The results were analyzed for *p* = 0.05.

## 3. Results

Table 2 presents the board moisture content 24 h after pressing. It was relatively low, probably due to the low average moisture content of the mat (about 10.5%) and long pressing time. Nevertheless, this means that the moisture of the intermediate layer effectively transferred heat into the pressed mat. 

The study demonstrated a drop in the bending strength and modulus of elasticity irrespective of the thermoplastic type added to the face layers (Table 3). For both parameters, the drop was the greatest for the board supplemented with high-density polyethylene, and amounted to 5.5 N/mm^2^ for the bending strength and 860 N/mm^2^ for the modulus of elasticity. The observed changes in bending strength depended on the type of the thermoplastic, its weight and origin, and the form in which it was added to the mat. The values for PE were considerably higher than those for the other polymers. 

The analysis of the effect the origin of the thermoplastic exerted on the bending strength showed that the MOR of the boards made from secondary polymers (PP_R, LDPE_RZ, LDPE_RR) was higher than that of the boards made from virgin polymers (PP, LDPE). This was probably because the granulates of secondary polymers came in smaller sizes and the granules were more evenly spread over the board cross section. The MOR of the boards containing secondary polypropylene (PP_R) reached 24.6 MPa and was higher by 8.8% than that of the boards supplemented with virgin polypropylene (PP). The difference was statistically significant (Table 3). The MOR of the boards containing secondary polyethylene, LDPE_RZ and LDPE_RR, were 26.1 and 25.9 MPa, respectively, i.e., by 6.5% and 5.7% higher than of the boards enriched with virgin polyethylene. This time, the differences were insignificant (Table 3).

Further analysis demonstrated higher values of MOR for LDPE than HDPE, and for agglomerate than granulate. We also found that the lower melting point of the granulate was, the more favorable its effects were on the bending strength. However, Tukey′s test identified the changes in MOR resulting from replacing some of the lignocellulosic material with recycled LDPE as insignificant. Moreover, the bending strength of the boards containing LDPE granulate was not statistically lower than of those supplemented with LDPE agglomerate. The type of polymer significantly affected the modulus of elasticity. This was probably due to the considerably lower modulus of elasticity of the polymers themselves vs. the boards made from lignocellulosic materials. Of the polymers used, only PS had the modulus of elasticity comparable to the boards composed of wood chips or annual plant particles. In this case, the MOE reached 3100–3300 N/mm^2^ [48,49,50]. The modulus of elasticity was also clearly higher in those boards vs. the boards made from the remaining polymers, but it was still lower than the MOE of the boards made from pine chips. Nevertheless, when considering the parameters of the bending test provided in the EN 312-7 standard [51], the boards may be classified as P7 type.

The results obtained in this study are in general agreement with those reported by other researchers [52], who found that the mechanical strength of the boards made from straw and isocyanates was much lower than those made from wood under the same bonding conditions. Like with any other lignocellulosic fiber in reinforced plastic composites, the major concern when using straw is its relatively poor compatibility with hydrophobic thermoplastics, which leads to poor mechanical properties of the boards [36]. To improve the compatibility of straw and water-based synthetic resin or thermoplastic polymers, special pretreatments of straw or polymers are necessary, such as steam, cold plasma, corona, or chemical treatment, the mechanical pulverization of a wax layer, or the addition of external compatibilizers [22,33,53,54,55,56].

The thermoplastics should only slightly affect tensile strength perpendicular to the board plane, as they were added to the face layers responsible for properties determined in the bending test. However, Table 4 shows significant differences in these values. Post hoc analysis revealed two levels of tensile strength perpendicular to the board plane, with the first ranging around 0.32–0.38 N/mm^2^ and the second around 0.38–0.42 N/mm^2^. The values within these levels were highly variable, probably because in numerous samples, and in many cases in most of them, only the external layer was damaged. It was only in a few samples the damage reached deeper layers, mainly the core layer. Therefore, we also provided maximum values which, except for the PP board, we found for damage of the core layer. The low quality of the subsurface layers was most probably due to overheating of the external layers during the long pressing time. This should be taken into account while designing future studies. Still, the maximum values indicate a possibility of producing this type of board with a strength exceeding 0.45 N/mm^2^. The improvement in the internal bond strength of WPCs vs. the control boards can be explained by the fact that, during hot pressing, some of the melted thermoplastic penetrates into the core layer. Then, the voids and spaces among the wood particles are filled with a melted thermoplastic polymer improving the adhesion between the particles.

For tensile strength perpendicular to the board plane determined after the boiling test, the share and type of the thermoplastic should not considerably affect this value that ranged from 0.10 to 0.13 N/mm^2^. Although the statistical analysis indicated some significant differences between the individual boards, they should be recognized similar.

As the thermoplastic polymers are good barriers to water due to their hydrophobic character, adding them to the outer and intermediate layers helps to significantly reduce thickness swelling and water absorption. All the boards containing the polymers in their external layers showed smaller thickness swelling than the reference ones. The differences ranged from 8.5% to nearly 22% (Table 5). Except for the HDPE boards, the improvement in thickness swelling was greater than the polymer share that was about 10%. The reduction in board swelling was greater for polymers with a lower softening point, which is why we achieved the best effects for recycled LDPE and polystyrene. The thickness swelling of boards containing these polymers was significantly lower than of the remaining ones. The degree of hydrophobic protection provided by the investigated polymers was better visible in the TS-X test, which involved immersing the boards in water for two hours while protecting their narrow surfaces with liquid foil. The results were highly variable within the experimental groups due to the degradation of the foil protecting narrow surfaces and uneven distribution of the polymers in the external layers. For the polymers containing boards, they were nearly two times lower than the control boards. Moreover, the changes were more favorable for the boards containing LDPE agglomerate, i.e., easily softening the polymer in their external layers.

**Figure 3:**
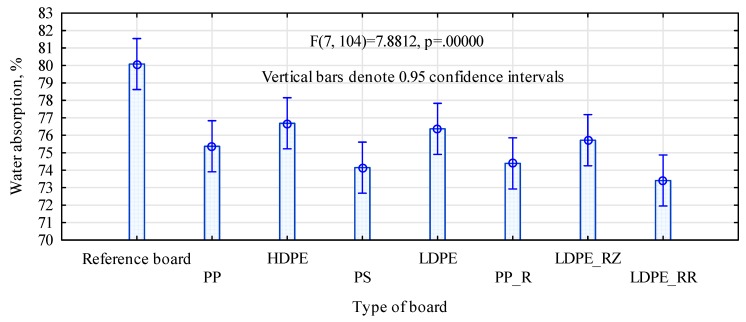
Water absorption in boards made from different materials.

The absorbability tests showed the boards absorbed from 73% to 80% water (Figure 3). The highest absorbability was determined for the reference board and the lowest for the board containing recycled LDPE which originated from pink foil. Thus, our zero hypothesis, assuming that all produced boards absorb similar amounts of water, shall be discarded. Post hoc tests indicated that the boards enriched with the polymers in their external layers absorbed less water than the reference boards. HSD Tukey′s test also indicated significant differences in absorbability for LDPE_RR and HDPE boards, but this was not confirmed by the Bonferroni test. The boards with a 30% share of polymers in their face layers absorbed only by 4%–7% less water than the control, thus indicating that this content of the polymer did not significantly limit water absorption by the lignocellulosic material. 

## 4. Conclusions

The introduction of thermoplastics to the face layers of the mat significantly changed the mechanical properties and water resistance of the experimental boards.

The most favorable results for bending strength and modulus of elasticity were achieved for boards enriched with polystyrene and recycled polyethylene. The boards containing polystyrene showed a smaller decrease in bending strength and maintained a very high modulus of elasticity, while the boards supplemented with recycled polyethylene featured a bending strength similar to the reference boards but low modulus of elasticity.

The tensile strength perpendicular to the board plane increased considerably irrespective of the origin of the thermoplastics added to the face layers, but the best results were achieved for polyethylene recycled from yellow foil and low-density polyethylene.

The virgin thermoplastics provided more favorable tensile strength values after the boiling test than recycled thermoplastics, which is why the boards of the greatest strength were those containing polystyrene and virgin polyethylene of lower density.

Irrespective of their type, thermoplastics added to the face layers favorably reduced hydrophobic properties of the boards, i.e., thickness swelling and water absorption.

## Figures and Tables

**Table 1:** Basic properties of the investigated thermoplastics.

Thermoplastic Type	Symbol	Thermoplastic Form	Density (g/cm^3^)	Softening Point (Vicata (A50))	MFR (Melt Flow Index)
Polypropylene, impact copolymer	**PP**	Granulate	0.9	150 °C	4 g/10 min (230 °C/2.16 kg)
High-density polyethylene	**HDPE**	Granulate	0.952	125 °C	0.3 g/10 min (190 °C/2.16 kg)
Polystyrene	**PS**	Granulate	1.5	90 °C	6 g/min (200 °C/5 kg)
Low-density polyethylene	**LDPE**	Granulate	0.923	91 °C	1.5 g/10 min (190 °C/2.16 kg)
Polypropylene from reusable packaging	**PP_R**	Agglomerate	0.54 *	nd	nd
Low-density polyethylene from yellow plastic	**LDPE_RZ**	Agglomerate	0.37 *	nd	nd
Low-density polyethylene from pink plastic	**LDPE_RR**	Agglomerate	0.38 *	nd	nd

* bulk density; nd not determined.

**Table 2:** Board moisture content after pressing.

Board Type (Thermoplastic)	Moisture Content		
	*x*, % *	SD, %	υ, %
**Reference Board**	3.70	0.06	1.69
**PP**	3.13	0.09	2.99
**HDPE**	3.29	0.22	6.81
**PS**	3.07	0.04	1.36
**LDPE**	2.92	0.13	4.51
**PP_R**	3.16	0.06	1.96
**LDPE_RZ**	2.90	0.16	5.59
**LDPE_RR**	3.00	0.11	3.56

* *x*—mean; SD—standard deviation; υ—coefficient of variation.

**Table 3:** The effects of thermoplastics on bending strength and modulus of elasticity.

Board type (Thermoplastic)	MOR			MOE		
	*x*, N/mm^2^ *	υ, %	HSD	*x*, N/mm^2^	υ, %	HSD
**Reference board**	26.5	5.2	e	3880	4.3	d
**PP**	22.6	5.7	a,b	3020	4.6	a,b
**HDPE**	21.0	4.7	a	2930	3.4	a
**PS**	23.4	8.9	b,c	3490	3.4	c
**LDPE**	24.5	6.2	c,d	3150	5.6	b
**PP_R**	24.6	8.6	c,d	3180	4.3	b
**LDPE_RZ**	26.1	3.3	d,e	3030	4.2	a,b
**LDPE_RR**	25.9	2.7	d,e	3020	2.5	a,b
ANOVA **	*F*(7, 88) = 20.440, *p* = 0.00000			*F*(7, 88) = 52.715, *p* = 0.0000		

* *x*—mean; υ—coefficient of variation, HSD—Tukey′s test; ** *F*—distribution; *p*—probability value.

**Table 4:** Effects of the thermoplastics on tensile strength perpendicular to the board plane.

Board Type (Thermoplastic)	IB				V100		
	*x*, N/mm^2^	υ, %	Max	HSD	*x*, N/mm^2^	υ, %	HSD
**Reference board**	0.33	17.8	0.43	A	0.10	10.1	A
**PP**	0.33	14.4	0.40	A	0.12	10.5	A,B
**HDPE**	0.32	10.0	0.44	A	0.12	13.1	B
**PS**	0.42	9.53	0.49	B	0.13	15.7	B
**LDPE**	0.40	9.35	0.45	B	0.13	7.6	B
**PP_R**	0.40	9.44	0.44	B	0.12	13.6	A,B
**LDPE_RZ**	0.41	12.1	0.48	B	0.13	11.5	B
**LDPE_RR**	0.38	12.7	0.45	A, B	0.12	13.7	A, B
**ANOVA**	*F*(7, 88) = 9.3263, *p* = 0.00000				*F*(7, 88) = 5.5786, *p* = 0.00002		

**Table 5:** Effects of the thermoplastics on board swelling after immersion in water.

Board Type (Thermoplastic)	TS			TS-X		
	*x*, %	υ, %	HSD	*x*, %	υ, %	HSD
**Reference board**	24.96	4.7	A	4.51	13.5	A
**PP**	21.99	3.7	B,C	2.36	20.0	B
**HDPE**	22.83	3.7	B	2.30	36.2	B
**PS**	19.51	4.4	F	1.64	32.0	B
**LDPE**	21.29	3.3	C,D	2.34	15.9	B
**PP_R**	20.41	5.2	D,E	1.99	35.0	B
**LDPE_RZ**	19.35	4.5	F	1.77	29.8	B
**LDPE_RR**	19.88	3.7	F	1.51	28.0	B
**ANOVA**	*F*(7, 88) = 55.929, *p* = 0.0000			*F*(7, 77) = 25.680, *p* = 0.0000		
